# YOLOv11-Lite architecture for wildlife detection from drone images

**DOI:** 10.3389/frai.2026.1777913

**Published:** 2026-03-24

**Authors:** Sherly Alphonse, Sahaya Beni Prathiba, Abhiram Sharma

**Affiliations:** 1 School of Computer Science and Engineering, Vellore Institute of Technology, Chennai, Tamil Nadu, India; 2 Centre for Cyber Physical Systems, School of Computer Science and Engineering, Vellore Institute of Technology, Chennai, Tamil Nadu, India

**Keywords:** animal, drone, UAV, wildlife, YOLO

## Abstract

**Introduction:**

Drones equipped with cameras are helpful in wildlife tracking. Deep learning has great potential for detecting wildlife, but is constrained by the challenge of detecting tiny objects, especially from higher altitudes.

**Methods:**

These limitations are addressed by an enhanced You Only Look Once 11 (YOLOv11-Lite) model. YOLOv11-Lite is a lightweight, edge-friendly variant of YOLOv11 that reduces computational complexity while maintaining high detection accuracy. Standard Convolution + Batch Normalization + SiLU (CBS) blocks are replaced with Depthwise-CBS units, which reduce the number of parameters and FLOPs. The enhanced version employs a Spatial Reasoning-Enhanced Coordinate Attention-based Simple Attention Module (CA-SimAM) for improved feature representation, Dynamic Sampling (DySample) for adaptive sampling, and a bounding-box IoU for accurate localization. The C2 block with the Parallel Split Attention (C2PSA) module is also replaced with a Ghost-ELAN block, as it enables ghost feature generation and multi-branch ELAN aggregation, achieving good performance with fewer computations.

**Results:**

The multiscale detection head aids in detecting smaller animals. The enhanced model achieves an mAP@0.5 of 98.5% and an mAP@0.5:0.95 of 94.7% on the WAID dataset.

**Discussion:**

The performance of the model is assessed through comparative tests, which demonstrate the superiority of the enhanced YOLOv11-Lite model over existing algorithms. The proposed approach supports UAV-based wildlife monitoring and improves detection performance and generalization under real-world conditions.

## 1 Introduction

Monitoring wildlife is crucial for biodiversity conservation, and conventional wildlife monitoring techniques, such as ground surveys and manual aerial observations, have significant limitations. Recently, Unmanned Aerial Vehicles (UAVs) using high-resolution cameras have been used for wildlife surveillance with less disturbance to animals (Ivanova et al., 2022; Hodgson et al., 2018). Many deep learning-based approaches, such as Faster Region-based Convolutional Neural Network (R-CNN), Single Shot MultiBox Detector (SSD), and You Only Look Once (YOLO), have enhanced the automated detection of wildlife in aerial imagery (Ren et al., 2015; Liu et al., 2016; Jocher et al., 2020). Still, some drawbacks exist, like the aerial viewpoints appearing small and partially occluded with varying illumination conditions (Lu and Lu, 2023; Yang et al., 2023; Bakana et al., 2024). Current object detection techniques cannot overcome these challenges and demand high computational costs.

Despite significant advancements in deep learning, especially Convolutional Neural Network (CNN)-based approaches, problems remain in object detection (Wang et al., 2025a). The most intractable of these is small object detection, which is crucial in wildlife monitoring because of the high-altitude flight of the drones and extensive natural environments to be monitored. Deep learning models, such as YOLO variants, have demonstrated strong performance in real-time detection tasks due to their improved design and fast inference. Yet most highly accurate YOLO models are computationally intensive and not suitable for real-time deployment on UAV platforms due to power consumption, memory constraints, and latency. YOLOv11 has good backbone refinement, enhanced feature aggregation, and improved detection head design compared to earlier models like YOLOv8 and YOLOv7 (Wang et al., 2022; Jocher et al., 2023). YOLOv7, proposed by Wang et al., offers improved feature extraction, improving accuracy. YOLOv8 by Jocher et al. integrates anchor-free detection and optimized loss functions. This model is better for low-resolution targets. YOLOv11 has a stronger feature-extraction foundation and better real-time inference capabilities. Thus, YOLOv11 is suitable for lightweight structural optimization. The proposed YOLOv11-Lite framework is an enhanced version of the YOLOv11 baseline that reduces computational complexity and improves detection robustness. The conventional lightweight YOLO variants rely on channel pruning or depthwise convolution to reduce parameters (Mittal, 2024). Existing YOLO versions are not accurate for small and distant wildlife targets. To address this issue, an improved YOLOv11 model, YOLOv11-Lite, is introduced, incorporating a Spatial Reasoning-enhanced Coordinate Attention-based Simple Attention Module (CA-SiMam), Bounding Box IoU (Bbox IoU), and Dynamic Sampling (DySample) to enhance small-object detection in aerial imagery. These improvements are focused on small-object feature representation, bounding box optimization, and adaptive sampling strategies. The contributions of this study are signified as follows:

- An enhanced YOLOv11-Lite model is developed to identify wild animals in drone images, replacing heavy YOLOv11 components with lightweight blocks.
- The conventional CBS (Conv-BN-SiLU) layers in YOLOv11 are replaced with Depthwise CBS(DW-CBS) blocks, which reduce convolutional computation while improving spatial feature extraction.
- A spatial reasoning-enhanced Coordinate Attention-based SimAM(CA-SimAM) is used to extract the important information.
- Ghost-ELAN replaces heavy convolutional branches with lightweight operations, thereby minimizing parameters and FLOPs by 40–60%.
- The neck structure is redesigned with reduced channel widths.
- Light-weight DySampling improves performance without increasing the number of computations.
- The Bounding box-IoU helps in identifying the wild animals more accurately.
- There are substantial reductions in parameters, FLOPs, and memory usage while retaining strong mAP performance, enabling reliable real-time detection.
- The inclusion of the 160 × 160 very-small-object head enhances the performance, especially for tiny animals.

The rest of the paper is structured as follows. Related research is presented in Section 2, which summarizes previous studies on deep learning-based object detection and wildlife monitoring. The suggested methodology is explained in detail in Section 3, which covers the components used in the enhanced YOLOv11-Lite. The primary elements, like DW-CBS, Ghost ELAN, CA-SimAM, DySample, and Bbox IoU, and their mathematical formulations are covered in the same section. The results are covered in Section 4, along with benchmarking against current models, performance evaluation, and ablation studies. The exact section presents an ablation study that examines the effects of various improvements on detection performance. Finally, Section 5 concludes the study and discusses future improvements.

## 2 Related work

The significant advancements in UAV technology and deep learning have transformed wildlife monitoring from manual observation to an automated process. But there are several challenges, like scale variation, small object size, background camouflage, and viewpoint distortion. Axford et al. (2024), in their survey of deep learning methods for animal detection using UAV images, highlighted the use of lightweight, small-object-aware models. This study evaluates challenges, including occlusion and scale differences, that affect wildlife detection performance. They suggested the need for improved multi-scale feature representation to improve the detection accuracy. Also, Xu et al. (2024) reviewed the aerial and satellite-based animal detection techniques and indicated the importance of robust generalization across environments. They also highlighted the importance of adaptive attention mechanisms to improve reliability in wildlife surveillance. The thermal sensors can also be used, as Beaver et al. (2020) indicated that thermal UAV imagery enhances animal detectability, noting the challenges posed by temperature variations. The study indicates that thermal sensors can improve visibility more effectively than RGB imagery.

Xu et al. (2024) studied detection techniques for both aerial and satellite imagery to address challenges of scale variation and occlusion. They emphasize the need for more robust architecture to enhance reliability in wildlife monitoring using aerial and field images. Ali and Zhang (2024) provided a review of the YOLO family, describing its architectures and efficiency trade-offs. This study highlights the challenges of detecting small objects and handling complex backgrounds in aerial imagery. The lightweight models can be applied for edge and drone-based platforms as suggested by Mittal (2024). This study analyzes architectures that reduce model complexity without degrading accuracy. Furthermore, Tang et al. (2023) proposed UAV-based object detection with some adaptation for real-time constraints. Beaver et al. (2020) focused on the application of thermal imaging and emphasized the detection frameworks that use thermal data. Bakana et al. (2024) proposed a YOLO5-based model with architectural modifications to automate wildlife monitoring, overcoming the challenges of real-time environments. Kangunde et al. (2021) reviewed real-time drone control systems and their applications. They suggested that reliable real-time control and data acquisition are needed for aerial wildlife surveillance. Ivanova et al. (2022) surveyed the use of drones for wild animal monitoring. The study analyzed how aerial platforms support tracking under challenging situations. Madasamy et al. (2021) proposed an embedded system-based framework where a small drone is integrated with a YOLO model. The study was done in resource-constrained aerial platforms. Their research suggests the deployment of deep learning models in real-time applications. Li et al. (2023) suggested an intelligent monitoring system. They used convolutional neural network models and highlighted the potential of data-driven detection systems in surveillance tasks. Roy et al. (2023) focused on “WilDect-YOLO: An Efficient and Robust Computer Vision-Based Accurate Object Localization Model for Automated Endangered Wildlife Detection”. But the model was not thoroughly tested across diverse habitats or species, so an important question remains about its generality across different ecological settings. This model enhances the robustness under different scales and environmental conditions.

Yang et al. (2023) proposed an improved algorithm of forest wildlife detection using YOLOv5s, aiming to improve the monitoring accuracy in complex forest environments. Ma and Yang (2022) explained a YOLOv5-based network for automated animal monitoring under diverse field conditions. Lu and Lu (2023) proposed a WD-YOLO for the detection of wildlife and the effective handling of multi-scale and overlapping targets. Liu et al. (2016) proposed an object detection framework using the YOLO network to provide effective real-time performance in many applications. In literature, authors have also proposed a few optimizations, including improved anchor box selection and a new loss function, which have enhanced detection accuracy at high frame rates (Liu et al., 2018). Wang et al. (2025a) proposed an advanced YOLO, a more advanced vibratory position detection algorithm derived from YOLOv11, with improved feature extraction methods to enhance detection performance in complicated environments. Their research proves that the YOLO architecture can be optimized to significantly improve object localization and classification in challenging situations. YOLO-based detectors can be further enhanced to address challenges in small-object detection and computational efficiency.

Min et al. (2023) proposed YOLO-DCTI for small object detection in remote sensing images. The method combines a Contextual Transformer (CoT) with global residual and local fusion mechanisms. Also, the decoupled contextual transformer detection head helps to achieve a better balance between speed and accuracy. STF-YOLO (Hui et al., 2024) incorporates contextual attention mechanisms that improve feature representation for small and occluded targets. This method combines a SwinTransformer-CNN hybrid structure (STRCN), a lightweight classifier (CNeB), SimAM attention, and a novel detection head (CWDHead). Also, a new data augmentation strategy improves accuracy on the VisDrone dataset. Lightweight YOLO architectures, such as LightUAV-YOLO (Lyu et al., 2025), have shown that efficient convolutional designs reduce model complexity while maintaining high accuracy. The authors (Gasimov, 2025) suggested a lightweight framework for night-time images with thermal-RGB fusion. The system uses a dual-branch CNN backbone and an adaptive sensor selection. There are some surveys on small object detection in remote sensing (Gui et al., 2024) that provide insights into different training strategies applicable to wildlife detection. These studies collectively motivate the development of a YOLOv11-Lite architecture optimized for accurate and efficient wildlife detection from UAV imagery. The limitations of the existing studies are given as follows:

- Existing models show poor performance while detecting small and distant animals.
- Existing approaches lack robust multi-scale feature representation.
- Existing models exhibit degraded performance, particularly in complex backgrounds and partial occlusions.

The current YOLO-based architectures perform better in real-time object detection, but often compromise small-object detection performance. In wildlife drone imagery, animals are often visible as small objects against complex backgrounds, which makes accurate detection challenging. Also, attention mechanisms such as Parallel split attention (PSA) have high computational overhead, which limits deployment in real-time environments. Therefore, a lightweight yet more accurate model for high-resolution small-object detection is needed. This gap is addressed by the enhanced YOLOv11-Lite architecture, which incorporates DW-CBS, Ghost ELAN, spatial reasoning, CA-SimAM, DySample, and Bbox-IoU. The comparison of the YOLOv11-Lite with the existing lightweight architectures is given in Table 1. The comparison of the YOLOv11-Lite and YOLOv11 is given in Table 2. Although a few approaches in the literature emphasize parameter reduction, an integrated approach that improves both efficiency and spatial sensitivity for wildlife drone imagery remains limited. Therefore, the YOLOv11-Lite framework re-engineers the baseline YOLOv11 architecture. The YOLOv11-Lite performs well on wildlife images as a lightweight, edge-friendly variant of YOLOv11, designed to achieve high detection performance. The Feature Pyramid Network-Path Aggregation Network(FPN-PAN) in the YOLOv11-Lite structure improves bidirectional multi-scale feature fusion. The advantages of each component are given as follows:

- **DW-CBS:** Reduces computational complexity while preserving feature representation.
- **Ghost-ELAN:** Minimizes channel redundancy and improves the efficiency during feature extraction.
- **Supplementary High-Resolution Head:** Enhances small-object detection at fine scales.
- **CA-SimAM:** Enhanced positional sensitivity using direction-aware channel attention.
- **Reduced neck channels:** Helps in the reduction of channels and total number of parameters.
- **DySample:** Applies adaptive upsampling, which enhances multi-scale feature alignment.
- **Bbox-IoU:** Enhances localization precision using bounding box regression.

**Table 1:** Comparison of the YOLOv11-Lite and other lightweight YOLO architectures.

**Model**	**Strategy**	**Attention**	**Dynamic upsampling**	**Neck optimization**	**Design**
YOLOv5-Nano; (Jocher et al., 2020)	Depth/width scaling	×	×	×	×
YOLOv7-Tiny; (Wang et al. 2022)	Structural Re-parameterization	×	×	×	×
Ghost-YOLO; (Han et al. 2020)	Ghost Modules	✓	×	Partial	×
Mobile-YOLO; (Howard et al. 2019)	Mobile Backbone	✓	×	✓	×
**YOLOv11-Lite**	DW-CBS + Ghost	✓ (CA-SimAM)	✓ (DySample)	✓ (Reduced Channels)	✓ (UAV Wildlife)

**Table 2:** Differences between YOLOv11 and YOLOv11-Lite.

**Component**	**Baseline YOLOv11**	**YOLOv11-Lite**	**Strategy**
Backbone structure	Standard YOLOv11 backbone	Same backbone	Retained
Loss function	IoU-based localization loss	BBox-IoU retained	Retained
Neck feature fusion	PAN/FPN with standard channel width	Reduced neck channel width	Re-engineered
C2PSA module	C2PSA	Substituted with Ghost-ELAN block	Re-engineered
Convolution block	Standard CBS (Conv-BN-SiLU)	DW-CBS	Re-engineered
Upsampling	Bilinear / Nearest Upsample	DySample	Re-engineered
Attention mechanism	PSA	CA-SimAM attention module	Introduced in this study
Small object detection	Three-scale detection heads	Supplementary 160 × 160 small-object detection head	Introduced in this study
Model complexity	Baseline parameter count and FLOPs	Reduced parameters and FLOPs	Performance improvement

The overall architecture and design are described in the following subsections.

## 3 Proposed methodology

The fundamental building blocks of the conventional YOLOv11 network are the Convolution + Batch Normalization + SiLU (CBS) blocks, resulting in stable and efficient feature processing. Furthermore, the Space-to-Depth Convolution present in the CBS blocks improves the detection of objects at low resolutions. The backbone of the model also includes the C3k2 (a cross-stage block with kernel size 2) block. These blocks are strategically placed throughout the network to maximize feature refinement (Li et al., 2023; Liu et al., 2016). The Spatial Pyramid Pooling-Fast(SPPF) block improves the receptive field of the network without an increase in computation. It also captures multiscale contextual information that helps improve the detection of small or distant objects.

To improve conventional YOLO models (Jocher et al., 2020; Wang et al., 2022; Jocher et al., 2023) for wildlife detection using drones, several enhancements have been proposed to boost small object detection, reduce background interference, and maintain real-time performance on embedded devices. The enhanced YOLOv11-Lite version is designed explicitly for wildlife monitoring. A key improvement in this model is the implementation of the CA-SimAM. The enhanced model introduces the Bounding Box Intersection over Union (IoU) to enhance localization accuracy, integrating shape consistency and spatial alignment.

**Figure 1:**
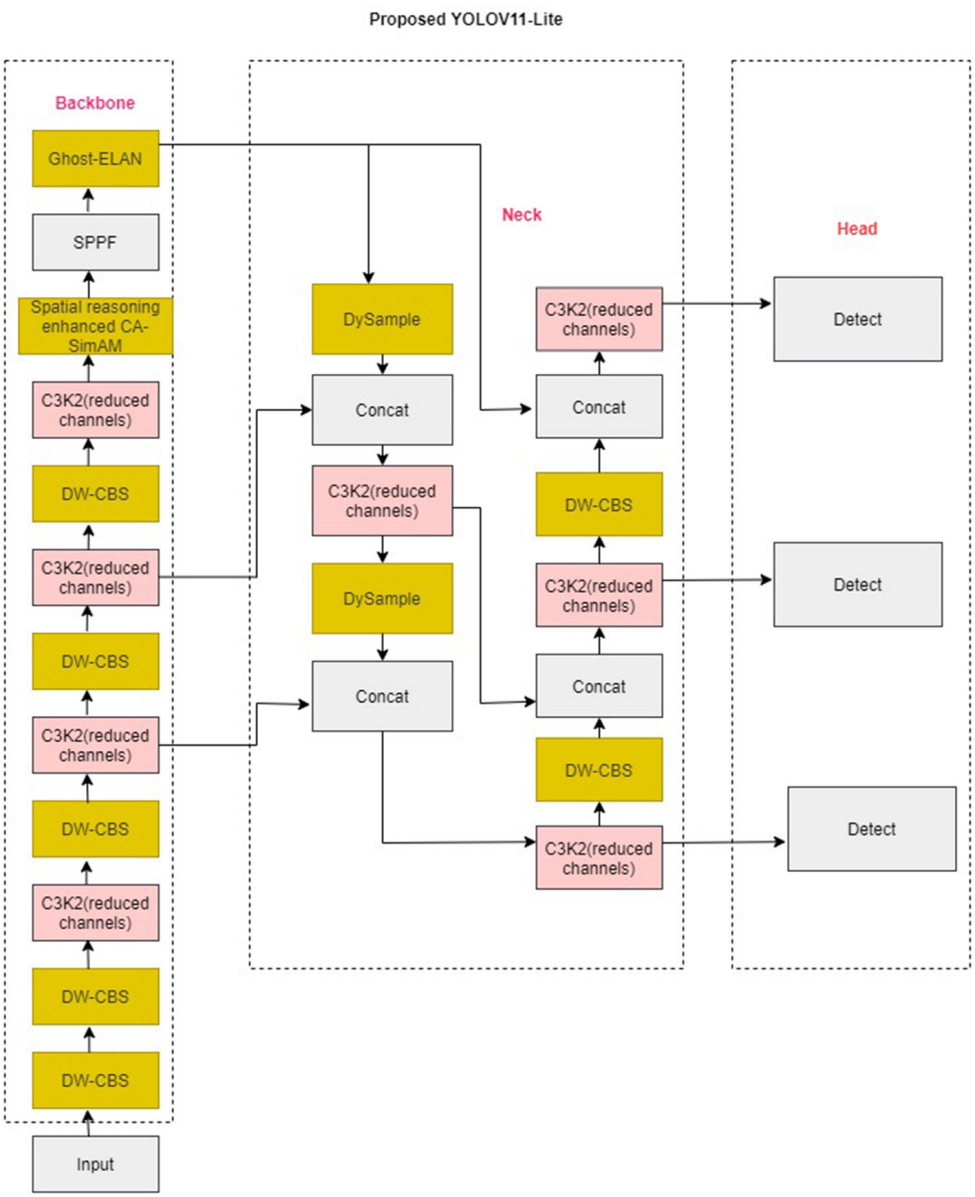
Architecture of the enhanced YOLOv11-lite (the colored blocks indicate the newly introduced or modified components).

YOLOv11-Lite is the lightweight version of Ultralytics (2024) and also preserves attention-enhanced feature modeling, making it suitable for identifying small wildlife. The architecture is illustrated in Figure 1. The enhanced model also substantially reduces parameter count and FLOPs by using depthwise convolutions, Ghost blocks, and a channel-reduced neck. The enhanced YOLOv11-Lite version replaces CBS blocks with depthwise separable DW-CBS blocks and also uses Ghost-ELAN modules instead of full ELAN blocks. Furthermore, the C2 block with Parallel Split Attention (C2PSA) modules is replaced with Ghost ELAN. This effectively balances the computational requirements with feature processing capabilities. The enhanced method is based on YOLOv11 with a Spatial reasoning-enhanced CA-SimAM to improve wild animal detection from higher altitudes. Coordinate Attention is a more effective method for enhancing fine-grained spatial understanding. Reduced neck channel complexity is used for lightweight processing, the bounding-box IoU formulation is employed for localization accuracy, and an enhanced multiscale detection head is added for improved scale-aware prediction. Each modification is introduced in a modular manner to ensure complementary performance gains while preserving real-time capability.

### 3.1 DW-CBS

Depthwise separable convolution block(DW-CBS) is used instead of the CBS in YOLOv11. It has a 3 × 3 depthwise convolution followed by a 1 × 1 pointwise convolution, along with batch normalization and SiLU activation.


$$\begin{array}{l} P_{c}\left(i,j\right)=\overset{k}{\underset{a=1}{\sum }}\overset{k}{\underset{b=1}{\sum }}X_{c}\left(i+a,j+b\right)K_{c}\left(a,b\right) \end{array}$$ (1)

**Where for a channel** *c* **:**

- *X*_*c*_ is the feature map given as input.
- *P*_*c*_(*i, j*) is the output obtained at spatial position (*i, j*).
- *K*_*c*_(*a, b*) is the depthwise convolution kernel.
- *i, j* are the spatial coordinates.
- *a, b* are the kernel indices.
- *k* is the kernel size.

### 3.2 Ghost block

This lightweight convolutional block is used in the enhanced YOLOv11-Lite, in which a subset of feature maps is generated by standard convolutions. Also, linear operations are used to produce the remaining maps, thereby reducing the number of parameters and FLOPs.


$$\begin{array}{l} G=\text{Concat}(\Phi_{1}\left(M\right),\Phi_{2}\left(M\right),\text{Ψ}\left(\Phi_{1}\left(M\right)\right),\text{Ψ}\left(\Phi_{2}\left(M\right)\right)) \end{array}$$ (2)

**Where:**

- *M* is the feature map given as input.
- Φ_1_(*M*), Φ_2_(*M*) are the feature branches computed using 1 × 1 convolutions.
- Ψ(·) : Ghost feature generator.

### 3.3 CA-SimAM block

A SimAM attention mechanism is applied to the feature maps, which are then modulated by a lightweight Coordinate Attention (CA) branch with a reduced channel ratio. CA-SimAM enhances the spatial reasoning in the improved model by combining direction-aware CA with pixel-level discriminative reasoning (SimAM). Compared to the standard attention mechanism (Squeeze-and-Excitation (SE)/Convolutional Block Attention Module (CBAM)), the Spatial Reasoning-Enhanced SimAM with CA has only lightweight 1 × 1 convolutions from CA, with a parameter reduction of approximately 10-15% at the YOLOv11-s scale. Moreover, CA-SimAM provides better global-local attention coupling, which improves small-object identification with minimal computational overhead.

**Figure 2:**
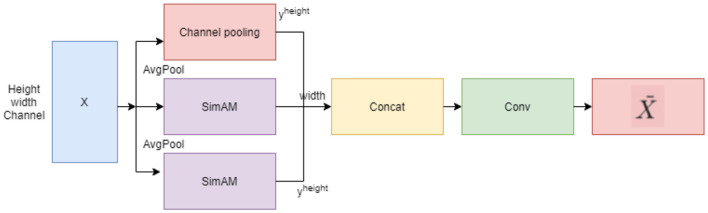
Spatial reasoning enhanced Coordinate Attention-based SimAM.

Continuously capturing the position information of this active neuron can capture the weight characteristics of the 3D feature of the image. SimAM proposes a unified weights approach to determine each neuron's weight by measuring linear separability. SimAM optimizes to determine neuron weights and can derive three-dimensional attention weights without additional parameters. The key advantage of SimAM (Hou et al., 2021; Yang et al., 2021) in wildlife detection lies in its spatial attention computation, as shown in Figure 2. For drone images, in which animals often appear as small objects within large landscapes, SimAM computes spatial attention weights using a variance-based approach. This method is especially valuable because it can distinguish wildlife from similar-looking environmental elements by measuring the spatial dependencies of features across different image regions. The mathematical formula of SimAM in the wildlife detection system can be expressed as:


$$\begin{array}{l} S=f\times \sigma \left(\text{λ}\times \left(𝔼\left(f^{2}\right)-𝔼{\left(f\right)}^{2}\right)+\mu \right) \end{array}$$ (3)

Where *E*(*f*) is the spatial average of features, λ is a learnable parameter, μ is a bias term, and σ is the sigmoid activation function.

Spatial reasoning is enhanced here by the application of coordinate attention. Coordinate Attention uses distinct global average pooling along the vertical (W) and horizontal (H) axes to represent location information. Horizontal pooling captures dependencies along the width and offers per-column descriptors. Vertical pooling provides per-row descriptors by capturing dependencies along the height dimension. These descriptors can be added to SimAM without introducing many parameters by using additive biasing or lightweight multiplicative modulation. The mathematical formula of Spatial reasoning-enhanced SimAM in the wildlife detection system can be expressed as:


$$\begin{array}{l} S=f\times \sigma \left(\text{λ}\cdot \left(𝔼\left(f^{2}\right)-𝔼{\left(f\right)}^{2}\right)+\alpha \cdot P_{b}+\beta \cdot P_{a}+\mu \right) \end{array}$$ (4)

Where *f* represents the input feature map from drone images, *E*(*f*) is the spatial average of features, α, β, λ are learnable parameters, μ is a bias term, *P*_*b*_ is the horizontal positional embedding obtained via global average pooling across the height dimension, *P*_*a*_ is the vertical positional embedding obtained via global average pooling across the width dimension, and σ is the sigmoid activation function.

The occlusions can affect the performance of the existing models. The enhanced model with spatial-reasoning-based CA-SimAM can distinguish animals more effectively than existing models. The disambiguation based on orientation becomes simpler in the enhanced model. Spatial reasoning-enhanced CA-SimAM helps in improving attention localization. The directional pooling in CA is represented as:


$$\begin{array}{l} g_{b}\left(c,b\right)=\frac{1}{A}\overset{A}{\underset{a=1}{\sum }}f\left(c,b,a\right),\;g_{a}\left(c,a\right)=\frac{1}{B}\overset{B}{\underset{b=1}{\sum }}f\left(c,b,a\right), \end{array}$$ (5)

Where A, B, and C represent the spatial width, height, and number of channels, $g_{b}\in {\mathbb{R}}^{C\times B\times 1}$ and $g_{a}\in {\mathbb{R}}^{C\times 1\times A}$ represent height- and width-aware context descriptors. The Shared 1 × 1 transformation is represented as


$$\begin{array}{l} u=\delta \left({\text{Conv}}_{1\times 1}\left(\left[g_{b},g_{a}\right]\right)\right), \end{array}$$ (6)

where δ(·) denotes the activation function and [·] is channel concatenation.

The Split and generate attention maps are indicated as:


$$\begin{array}{l} A_{b}=\sigma \left({\text{Conv}}_{1\times 1}\left(u_{b}\right)\right),\;A_{a}=\sigma \left({\text{Conv}}_{1\times 1}\left(u_{a}\right)\right), \end{array}$$ (7)

where $A_{b}\in {\mathbb{R}}^{C\times B\times 1}$ and $A_{a}\in {\mathbb{R}}^{C\times 1\times A}$.

The final CA attention is:


$$\begin{array}{l} M_{\text{CA}}=M_{h}\cdot M_{w}. \end{array}$$ (8)

$$\begin{array}{l} z=f⊙M_{\text{SimAM}}⊙M_{\text{CA}}, \end{array}$$ (9)

Where *M*_*S*_*imAM* denotes the attention map generated by SimAM, and ⊙ denotes element-wise multiplication.

By combining global coordinate priors with pixel-level contrast reasoning, the CA-SimAM produces a spatial attention map that focuses on true wildlife regions by suppressing background clutter, while improving fine-grained spatial understanding in UAV-based wildlife detection.

### 3.4 Reduced neck channels

Conventional YOLOv11 models use 256, 512, 1,024 channels for P3/P4/P5 feature fusion. The enhanced lightweight version uses 192, 384, 768. All subsequent fusion and 3 × 3 convolution layers have reduced widths. Neck channels are reduced from 256, 512, 1,024 to 128, 256, 384, and DySample is used for upsampling. While preserving CA-SimAM, attention is given to a reduced channel ratio in the Coordinate Attention branch. Thus, the parameter count is reduced while maintaining competitive detection mAP on WAID.

### 3.5 Dysample instead of upsampling

DySample improves upsampling accuracy without incurring significant parameter overhead. Using DySample instead of heavy upsampling layers reduces parameters while increasing accuracy for small wildlife detection. This design achieves better performance through a precise balance of elements, with a special focus on dynamic sampling and shape-aware detection mechanisms. Standard upsampling in the neck is replaced with a Dysample module, which enables content-aware upscaling with lower latency and improves multi-scale fusion efficiency for small-object detection.

**Figure 3:**
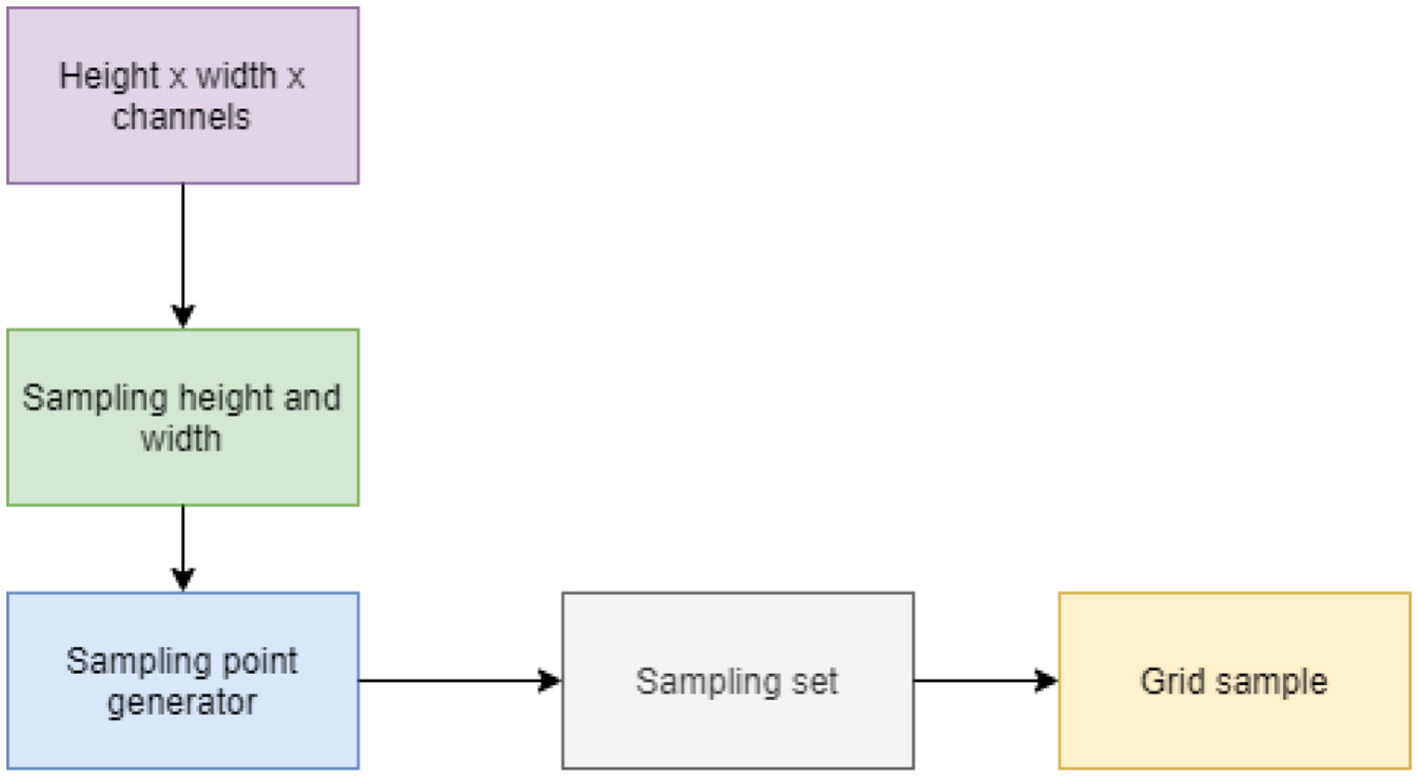
DySample process.

DySample is a lightweight and efficient upsampling technology. DySample does not require high-resolution features. The system intentionally selects points throughout the image for feature extraction. This module helps overcome issues specific to drone photography, such as perspective distortion and changes in lighting. It also uses adaptive sampling techniques to focus on areas of interest. The framework of DySample (Liu et al., 2023) is illustrated in Figure 3.

Finally, a grid sample is generated, and the grid arranges the sampling points into an organized pattern that allows for parallel processing and preserves spatial relationships among detected objects. This helps with strong object detection while minimizing computational resource consumption.

### 3.6 Bounding-box IoU

**Figure 4:**
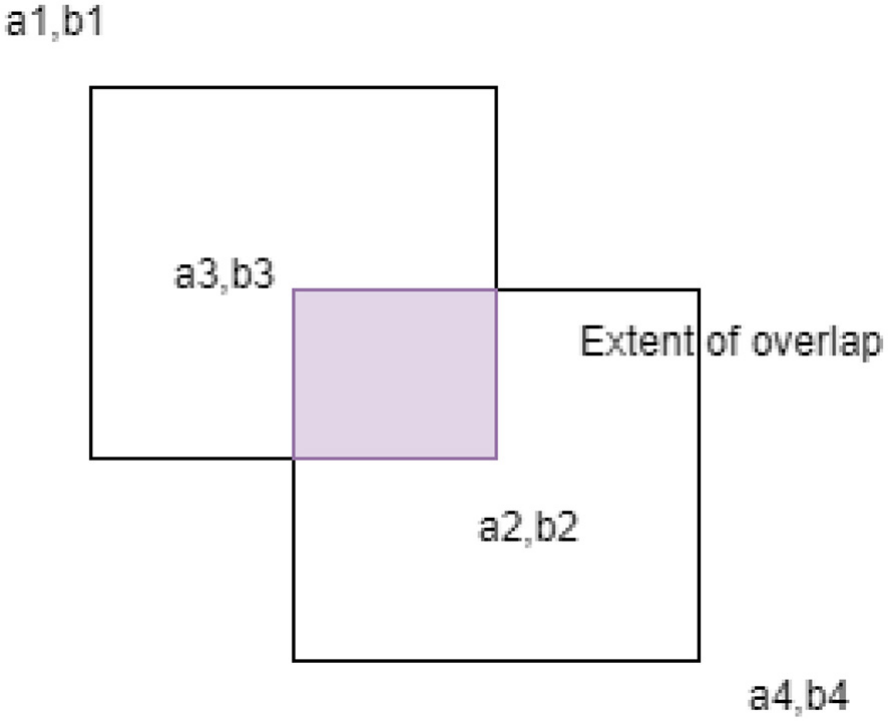
Intersection of two boxes.

The intersection region (Padilla et al., 2020), as in Figure 4, is defined by the overlap of Box 1 and Box 2, with its coordinates given by:


$$\begin{array}{l} \begin{array}{r} \text{left}=\max\left(a_{1},a_{3}\right)\\ \text{top}=\max\left(b_{1},b_{3}\right)\\ \text{right}=\min\left(a_{2},a_{4}\right)\\ \text{bottom}=\min\left(b_{2},b_{4}\right) \end{array} \end{array}$$ (10)

The final IOU is calculated as:


$$\begin{array}{l} \text{IoU}=\frac{{\text{Area}}_{\text{Intersection}}}{{\text{Area}}_{\text{Union}}} \end{array}$$ (11)

$$\begin{array}{l} {\text{Area}}_{\text{Intersection}}=\max\left(0,\text{right}-\text{left}\right)\times \\ \;\max\left(0,\text{bottom}-\text{top}\right)\\ \;{\text{Area}}_{\text{Union}}={\text{Area}}_{1}+{\text{Area}}_{2}-{\text{Area}}_{\text{Intersection}} \end{array}$$

In drone image-based wildlife detection, IoU is essential for assessing how well the detected bounding box matches an animal's ground-truth location. The greater the IoU, the higher the detection precision. A low IoU indicates that the predicted bounding box does not closely align with the ground truth. IoU is also employed in Non-Max Suppression (NMS) to remove duplicate bounding boxes and retain only the most confident detection per animal. IoU is also an essential part of Non-Max Suppression (NMS), as it removes duplicate bounding boxes and retains only the most confident detections. Optimizing IoU will make wildlife monitoring systems based on drone-based deep learning models more accurate and efficient.

#### 3.6.1 Enhanced multiscale detection head

While the existing YOLO frameworks produce predictions at three scales, the enhanced architecture introduces an additional small-object detection head that operates at 160 × 160 resolution. This higher-resolution prediction branch helps improve the detection of low-contrast wildlife instances.

The bidirectional FPN-PAN feature fusion structure inherited from YOLOv7 is retained in the enhanced YOLOv11-Lite multi-scale object detector. The Feature Pyramid Network (FPN) upsamples deeper semantic features and merges them with shallower layers, which generates high-resolution feature maps with rich contextual information. This is helpful in wildlife detection where animals appear at vastly different scales or occupy only a few pixels.

The Path Aggregation Network (PAN) has a complementary bottom-up enhancement pathway. PAN propagates fine-grained spatial features from earlier stages back toward deeper layers. This bidirectional pyramidal design effectively combines high-level semantics with low-level spatial details, leading to more robust predictions across scale variations. The FPN-PAN combination, therefore, enhances accurate multi-scale detection, especially in cluttered natural environments.

**Figure 5:**
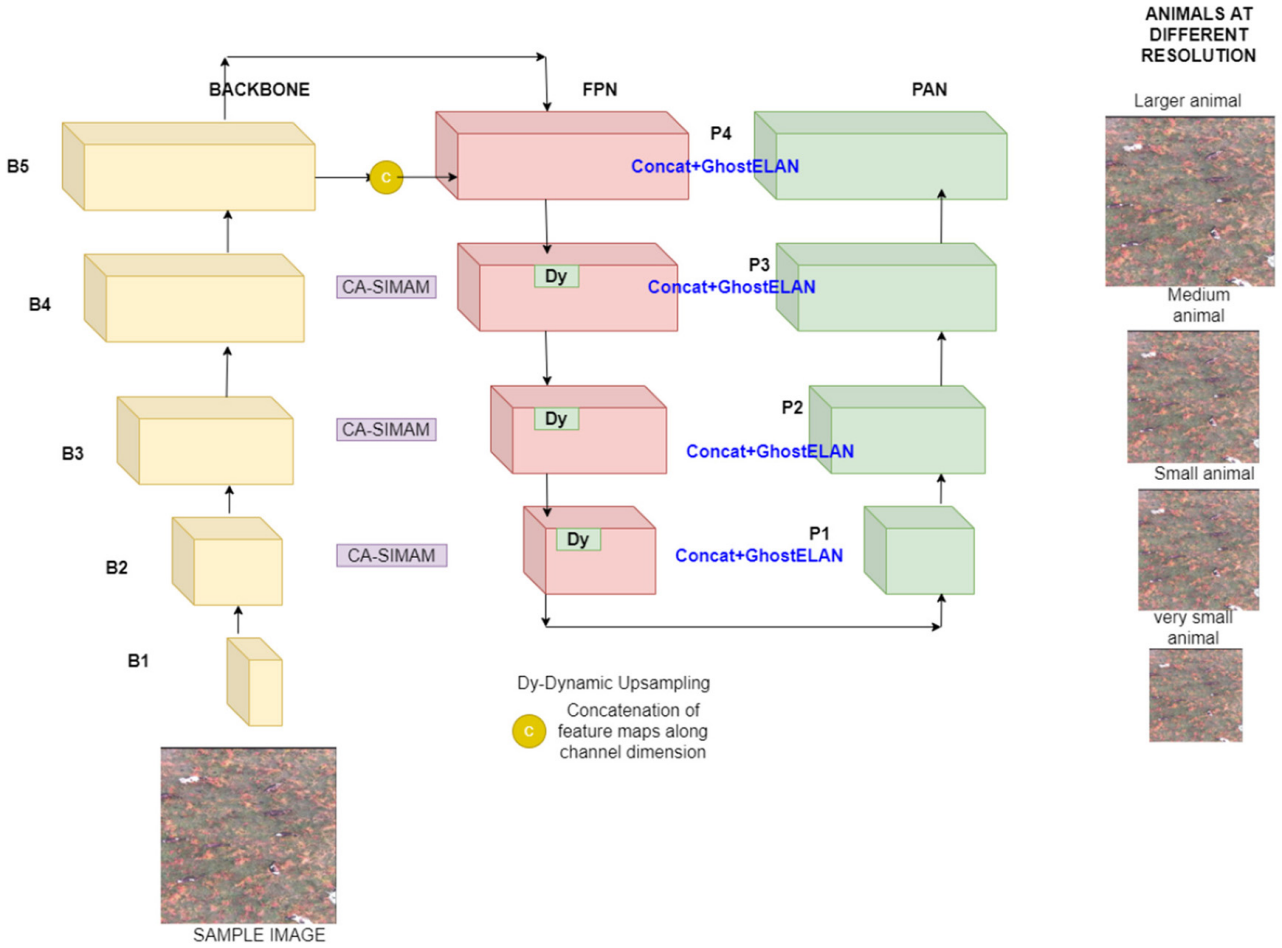
Multiscale detection head.

The FPN-PAN structure and the enhancements like DW-CBS, Ghost-ELAN, DySample upsampling, reduced neck channels, CA-SimAM attention, and an extra detection head (Lin et al., 2017) help in creating a revised head network, as illustrated in Figure 5.

## 4 Result analysis

The performance of the enhanced YOLOv11 is compared with that of different variants and state-of-the-art approaches. The enhanced approach improves the mAP with its multi-resolution approach. Several other datasets, as in [23–26], are also used in the experiments to analyze performance. A 5-fold stratified cross-validation strategy is used in the experiments.

**Figure 6:**
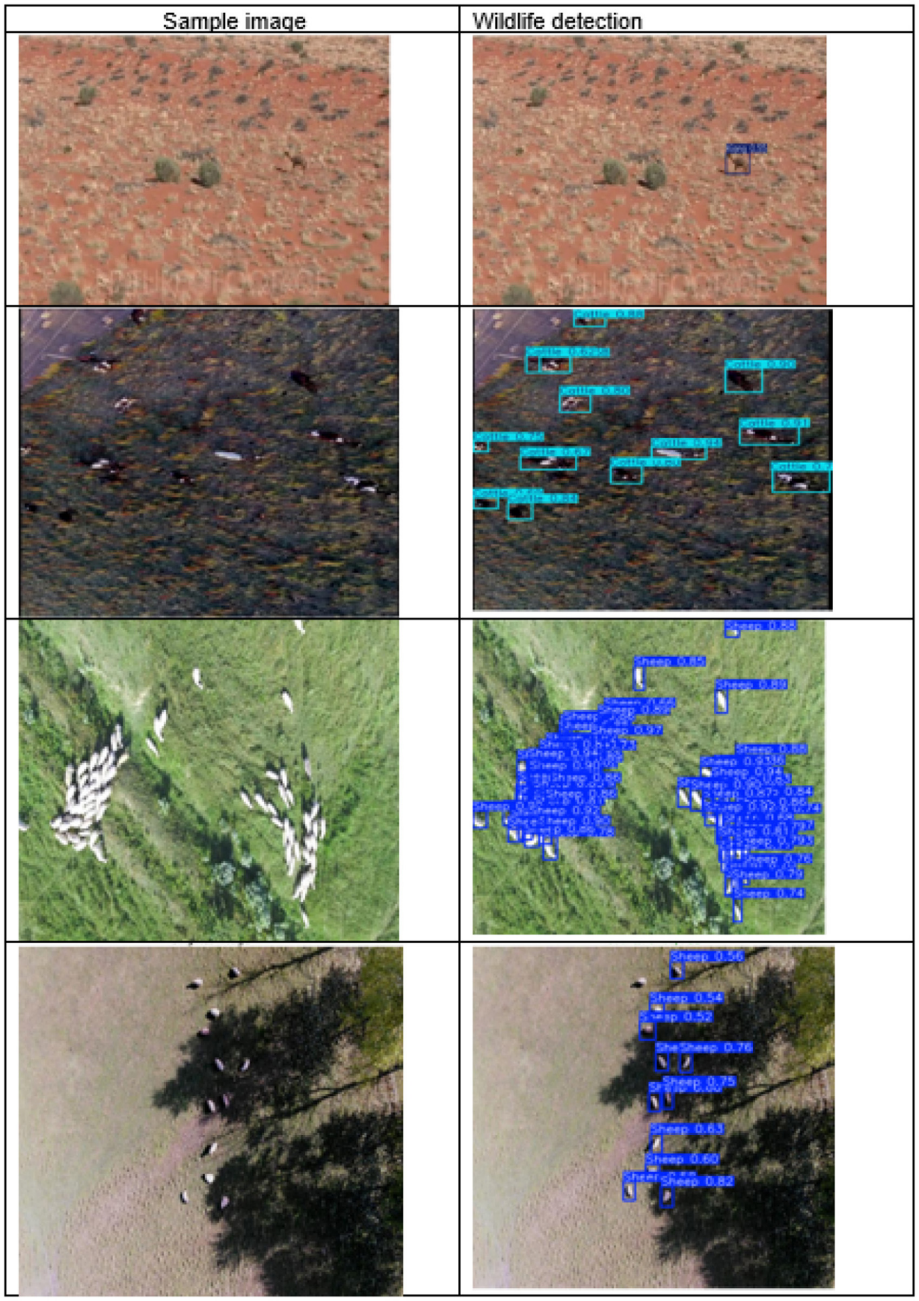
Wild life detection from sample image.

As the model undergoes training, its predictions become more accurate, as shown in Figure 6, with bounding boxes aligning more closely around individual objects. The results at the following stages present an improved, structured detection output with enhanced bounding boxes, thereby reducing misclassifications.

### 4.1 Training configuration

The PyTorch framework was used for the implementation, and all the models were trained under identical experimental settings. The following hyperparameters as in Table 3 are used:

**Table 3:** Hyperparameters used in YOLOv11-Lite.

**Category**	**Hyperparameter**	**Value**
Training	Input size	640 × 640
	Batch size	16
	Momentum	0.937
	Epochs	150
	Optimizer	Stochastic gradient descent
	Initial learning rate	0.01
	Learning rate scheduler	Cosine decay
	Weight decay	0.0005
Loss	Box loss gain	7.5
	Class loss gain	0.5
	Object loss gain	1.0
	IoU type	IoU (bounding-box IoU)
Augmentation	Mosaic	Enabled
	MixUp	Enabled
	Horizontal flip	0.5
Architecture	Depth multiple	0.33
	Neck channels	Reduced
	Width multiple	0.50
	Attention	CA + SIMAM
	Upsampling	DySample

The Mosaic augmentation is used with Random horizontal flipping, scaling, and translation, and Random rotation. The augmentation parameters are consistent across all the models used for comparison. Training was conducted on a workstation equipped with an NVIDIA RTX 4090 GPU (24 GB VRAM), an AMD Threadripper CPU, and 128 GB of RAM. Mixed precision (FP16) training is used to accelerate convergence and optimize memory utilization. During evaluation, standard inference configurations are used. A Confidence threshold of 0.25, Non-Maximum Suppression (NMS) IoU threshold of 0.7, and an evaluation image resolution of 640 × 640 is used. A 5-fold stratified cross-validation strategy was applied on the WAID dataset (2355 images across 10 wildlife classes). Stratification maintained proportional representation of each class within every fold.

For each fold, 1,884 images are used for training and 471 for validation. Detection performance was evaluated using mAP@0.5, mAP@0.5:0.95, Precision, and Recall. The proposed YOLOv11-Lite model is compared with the baseline models using a paired t-test across similar validation splits. The paired t-test indicated that the YOLOv11-Lite is statistically significant (*p* < 0.05).

### 4.2 Wildlife detection using WAID dataset

**Figure 7:**
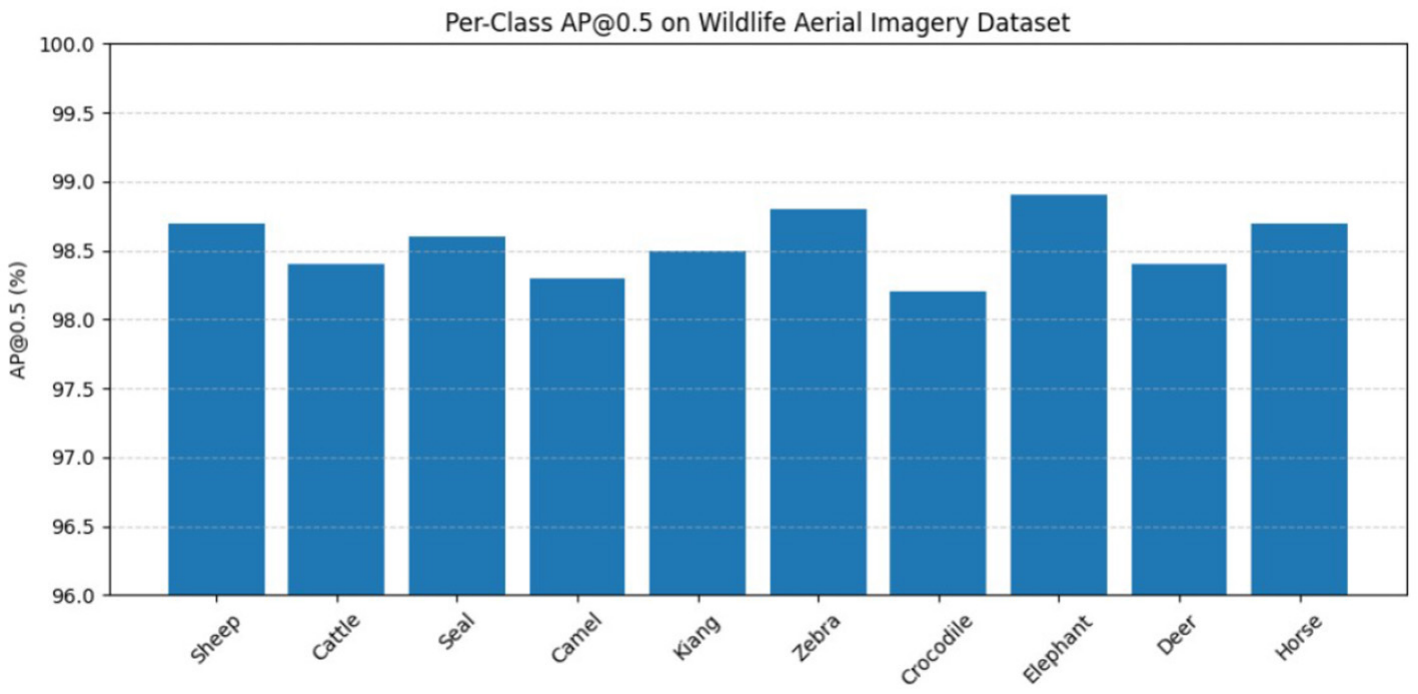
Per-class detection AP@0.5.

Figure 7 displays the good performance of the enhanced approach. Table 4 indicates the lightweight nature of YOLOv11-Lite, because of its reduction in the number of parameters, FLOPs, and model size compared to the other existing YOLO models. Also, the enhanced model has better inference speed and lower latency. Thus, the enhanced model outperforms existing models in both detection performance and computational efficiency.

**Table 4:** Efficiency analysis with the recent YOLO models on WAID.

**Model**	**Params (M)**	**FLOPs (G)**	**Model Size (MB)**	**FPS**	**Latency (ms)**
YOLOv8-s	11.2	28.6	21.4	83	12.3
YOLOv8-n	3.3	8.7	6.1	82	6.3
YOLOv11	9.6	24.3	18.5	96	10.3
**YOLOv11-Lite**	**5.1**	**12.9**	**9.6**	**145**	**6.9**

The values indicated using boldface represents the best values for the corresponding performance metric.

**Figure 8:**
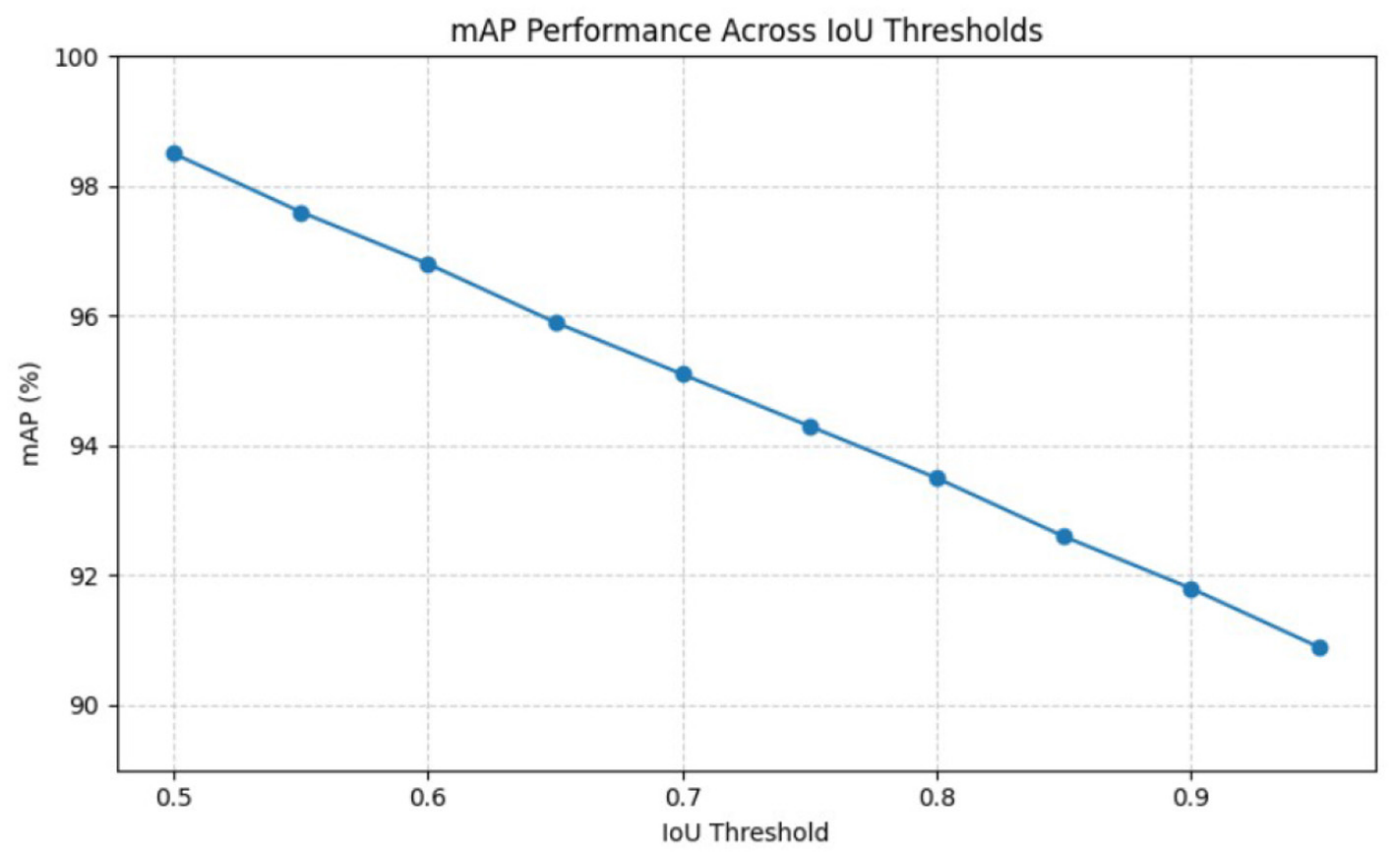
mAP performance across IoU thresholds.

Table 5 evaluates the performance of YOLOv11-Lite on challenging small and partially occluded wildlife instances. The animals are grouped into small, medium, and large categories based on their bounding box areas, while occluded instances are identified using visible-area estimation, derived from bounding-box overlaps. The enhanced YOLOv11-Lite attains high robustness even for occluded animals. The 98.5% mAP@0.5 and 94.7% mAP@0.5:0.95, as shown in Figure 8, indicate good performance under different backgrounds and lighting conditions. These improvements can be attributed to CA-SimAM, which enhances discriminative feature representation, and DySample, which refines multi-scale feature sampling. Overall, the results confirm the suitability of YOLOv11-Lite for real-world wildlife monitoring scenarios.

**Table 5:** Performance comparison with baseline for small and occluded wildlife animals.

**Type**	**Model**	**Precision**	**Recall**	**F1-score**	**mAP@0.5**
Small animals	YOLOv11	0.95	0.93	0.94	0.95
	**YOLOv11-Lite**	**0.98**	**0.97**	**0.98**	**0.985**
Occluded animals	YOLOv11	0.94	0.92	0.93	0.94
	**YOLOv11-Lite**	**0.97**	**0.96**	**0.96**	**0.98**

The values indicated using boldface represents the best values for the corresponding performance metric.

### 4.3 Ablation study on WAID dataset

The ablation study in Table 6 evaluates the contribution of each module to the overall performance of YOLOv11-Lite. Introducing depthwise CBS significantly reduces the parameter count while maintaining better detection. The addition of CA-SimAM improves feature discrimination, leading to higher mAP, particularly for small and occluded wildlife objects. DySample further enhances localization accuracy by refining the sampling of multi-scale features. The full YOLOv11-Lite model achieves the best performance with the lowest parameter count, validating the effectiveness of the enhanced lightweight design.

**Table 6:** Ablation study using WAID dataset.

**Model**	**DW-CBS**	**CS**	**DS**	**GE**	**MSH**	**mAP@0.5**	**Params (M)**
YOLOv11 (Conventional)	✗	✗	✗	✗	✗	93.2	9.6
+ DW-CBS	✓	✗	✗	✗	✗	94.2	7.6
+ DW-CBS + CA-SimAM(CS)	✓	✓	✗	✗	✗	95.4	7.5
+ DW-CBS + CA-SimAM + DySample(DS)	✓	✓	✓	✗	✗	96.5	7.7
+ DW-CBS + CA-SimAM + DySample + Ghost-ELAN(GE)	✓	✓	✓	✓	✗	98.0	6.2
**YOLOv11-Lite [enhanced with Multiscale Detection Head (MSH)]**	✓	✓	✓	✓	✓	**98.5**	**5.1**

The values indicated using boldface represents the best values for the corresponding performance metric.

### 4.4 Heatmap Visualization

**Figure 9:**
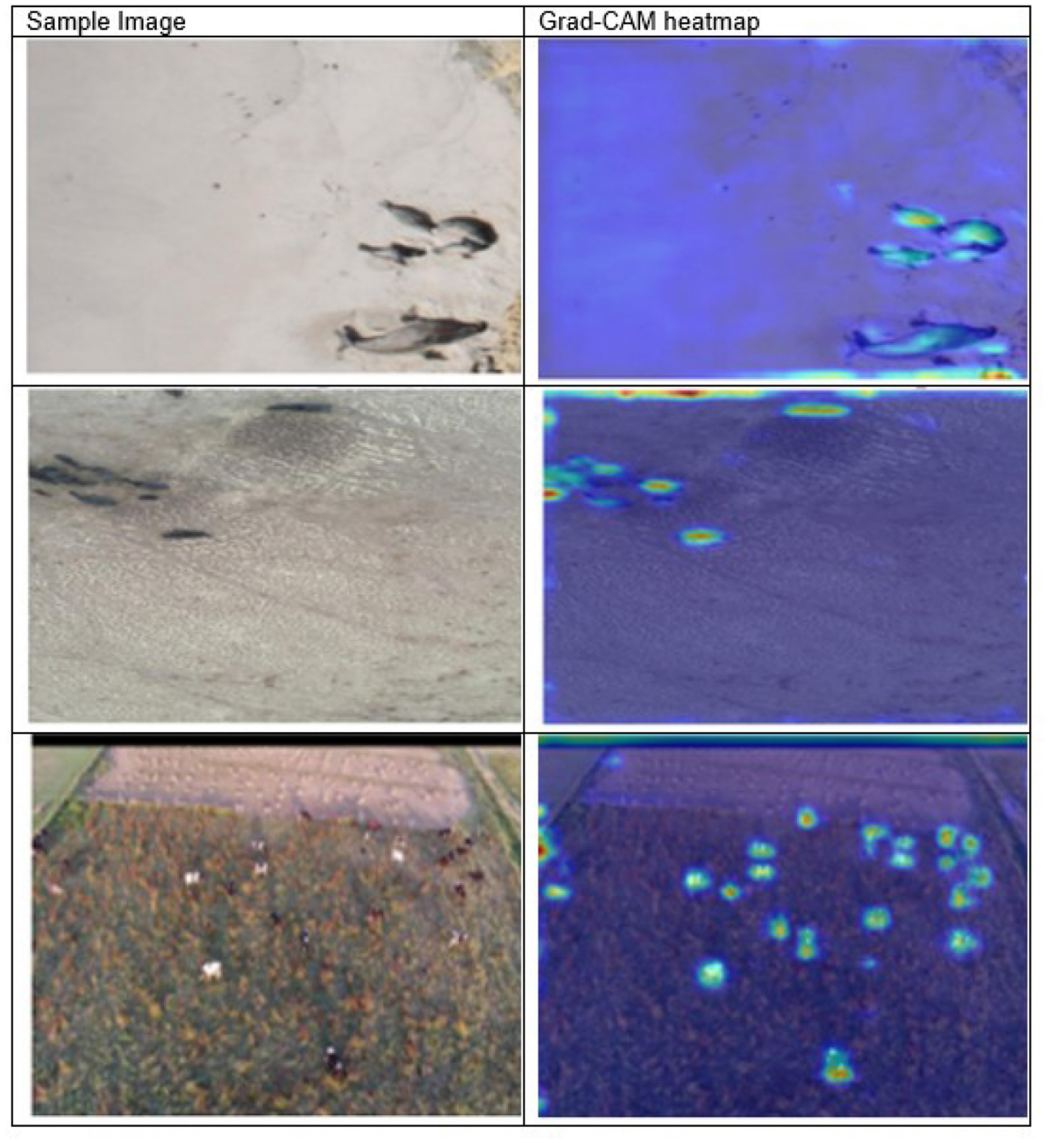
Grad-CAM heatmaps.

In the Figure 9, the Grad-CAM visualizations have warmer colors like red and yellow, indicating the areas of higher contribution, and the color blue indicates areas that have little influence. The target wildlife regions are indicated using red and yellow. Background regions are indicated in blue, representing irrelevant features. Thus, the enhanced model focuses on important object cues rather than irrelevant background patterns.

### 4.5 Comparison with different existing models using WAID

The utilization of DySample, which refers to Dynamic Sampling-based Upsampling, helps in wild animal localization. This also helps detect small and obstructed animals. Due to the DySample, the CA-SimAM and Multi-scale detection head, the YOLOv11-Lite model performs better than other conventional models. As such, it can be concluded that the enhanced YOLOv11-Lite is the best choice for real-time aerial monitoring and assisting conservation efforts, particularly in cases where speed and accuracy are of the utmost concern.

Table 7 compares YOLOv11-Lite with different object detection models in the literature on the WAID dataset. Two-stage detectors, such as Faster R-CNN, achieve good mAP but have significant drawbacks, including high computational cost and low inference speed. Conventional YOLO models are larger. The enhanced model achieves better performance with a 98.5% mAP@0.5 and 94.7% mAP@0.5:0.95 on the WAID dataset, while maintaining a compact model size and real-time inference speed of 145 FPS. Therefore, the enhanced lightweight architecture is effective for drone-based wildlife detection. Statistical significance testing between the enhanced YOLOv11-Lite and conventional methods was done with a paired t-test at a 95% confidence level. The mAP@0.5 on the WAID dataset by the enhanced approach is statistically significant (*p* < 0.05). Table 8 displays the best performance of the enhanced method compared to the benchmarks seen in the literature.

**Table 7:** Performance analysis using different object detection models on the WAID dataset.

**Model**	**Backbone**	**Params(M)**	**mAP@0.5**	**FPS**
Faster R-CNN; (Ren et al. 2015)	ResNet-50	41.1	89.3	18
SSD; (Liu et al. 2016)	VGG-16	26.2	87.5	45
YOLOv5-s; (Jocher et al. 2020)	CSPDarknet	7.6	93.7	120
YOLOv7; (Wang et al. 2022)	E-ELAN	36.8	95.5	70
YOLOv8-s; (Jocher et al. 2023)	CSPDarknet	11.2	96.3	82
YOLOv11; (Wang et al. 2025b)	ELAN	9.6	96.9	96
**YOLOv11-Lite**	Ghost-ELAN	**5.1**	**98.5**	**145**

The values indicated using boldface represents the best values for the corresponding performance metric.

**Table 8:** Comparison with state-of-the-art methods evaluated on the WAID dataset.

**Method**	**Backbone**	**mAP@0.5 (%)**	**Acc. (%)**	**Classes**
Faster R-CNN; (Mou et al. 2023)	ResNet-50	77.9	-	6
SSD; (Mou et al. 2023)	VGG-16	73.9	-	6
YOLOv5; (Mou et al. 2023)	CSPDarknet53	95.6	-	6
YOLOv7; (Mou et al. 2023)	CSPDarknet53	97.2	-	6
YOLOv8; (Mou et al. 2023)	CSPDarknet53	95.8	-	6
Yolov8; (Vaiyapuri et al. 2025)	EfficientNetB3	-	96.4	3
**YOLOv11-Lite**	Ghost-ELAN + CA-SimAM	**98.5**	-	10

The values indicated using boldface represents the best values for the corresponding performance metric.

### 4.6 Evaluation of the enhanced method across different datasets

The efficiency of the enhanced YOLO model was rigorously examined and evaluated against a diverse set of datasets, as shown in Table 9, to accurately assess its overall performance for object detection and segmentation. Under intense testing, the model was trained and subsequently evaluated on diverse datasets, including varying class distributions and image volumes. The study demonstrates that the model achieves a competitive mAP@0.5 across various datasets, validating its generalizability to new data. In datasets with more classes and more intricate, detailed backgrounds, the mAP@0.5 of the enhanced model is higher than that of other existing models.

**Table 9:** Evaluation of the YOLOv11-Lite for the different datasets tested.

**Dataset**	**Classes**	**Images**	**mAP@0.5 (%)**
WAID	10	2355	98.5
Animal-based drone; Roboflow (2023a)	4	1,082	91.3
Animal dataset; Roboflow (2023c)	6	3,154	90.7
Animal object detection; Roboflow (2023b)	4	2,692	92.2
Safari dataset; Roboflow (2024)	4	2,659	95.9
Cow dataset; University of Bristol Data Service (2020); (Andrew et al. 2021)	1	11,779	93.5

The YOLOv11-Lite achieves an mAP@0.5 of 98.5% on the WAID dataset, which has high annotation quality, relatively consistent aerial viewpoints, and balanced object representation. The performance does not solely reflect dataset-specific bias, as the cross-dataset evaluation shows that the model maintains stable generalization performance across different environmental conditions. Also, the risk of overfitting was addressed by training the model using data augmentation techniques, weight decay regularization, and early stopping criteria.

Due to variations in object scale, background complexity, and annotation differences across the different datasets used in Table 10, there is a drop in performance. But YOLOv11-Lite maintains a stable mAP@0.5 above 78% across all datasets, demonstrating strong cross-domain generalization capability and applicability to real-time environments.

**Table 10:** Cross-dataset evaluation.

**Training dataset**	**Testing dataset**	**Classes**	**mAP@0.5 (%)**
WAID	Animal-based drone	4	81.1
WAID	Animal Dataset	6	78.2
WAID	Animal object detection	4	84.3
WAID	Safari dataset	4	83.7
WAID	Cow dataset	1	84.2

## 5 Conclusion and future work

This study proposed a YOLOv11-Lite framework that improves performance in wildlife detection from UAV-based aerial imagery. This framework features a lightweight architectural design while still offering strong feature representation for small and distant animals, and it addresses challenges such as scale variation and computational constraints. From the experimental results, it is clear that the enhanced YOLOv11-Lite has better performance with less model complexity, demonstrating that it is well-suited for resource-constrained UAV platforms. This study balances mAP and efficiency. The performance gains achieved by YOLOv11-Lite are given as follows. YOLOv11-Lite reduces redundant feature extraction and preserves essential spatial information, thereby improving generalization. The supplementary detection head used is beneficial for detecting small wildlife objects by improving the recall of distant animals. This also helps in improving the mAP@0.5 and mAP@0.5:0.95. The performance improvement is primarily attributed to three architectural enhancements. The CA-SimAM helped in better localization of small and partially occluded animals. The DySample module helps with multi-scale feature aggregation, enhancing animal detection even under scale variation.

In the ablation study, as in Table 6, CA-SimAM, DySample, and GhostElan significantly contribute to improved performance. These modifications done in YOLOv11-Lite collectively enhance both detection accuracy and computational efficiency compared to baseline YOLO variants. The enhanced approach achieves an mAP above 90% on most datasets. But further validation under real-time scenarios is needed for universal deployment. Future research will focus on further optimizing YOLOv11-Lite to enhance real-time processing on resource-constrained embedded platforms. Investigating multi-modal data fusion, fusing thermal with regular aerial imagery, may also enhance detection in future studies under challenging environments like low-light or high-vegetation.

## Data Availability

The original contributions presented in the study are included in the article/Supplementary Material, further inquiries can be directed to the corresponding author.
